# The Timed 180° Turn Test for Assessing People with Hemiplegia from Chronic Stroke

**DOI:** 10.1155/2018/9629230

**Published:** 2018-01-15

**Authors:** Regan L. Robinson, Shamay S. M. Ng

**Affiliations:** Department of Rehabilitation Sciences, Faculty of Health and Social Sciences, The Hong Kong Polytechnic University, Hung Hom, Hong Kong

## Abstract

**Background:**

Turning is ubiquitous in activities of daily living. For people with hemiplegia, persistent impairments in strength, balance, and coordination will affect their ability to turn safely. Consequently, turning retraining should be addressed in rehabilitation programs. To measure turning for these individuals, a reliable clinical tool is required.

**Objective:**

To investigate (i) the intrarater, interrater, and test-retest reliability of the timed 180° turn test; (ii) the correlation of the timed 180° turn test with other measures of stroke-specific impairments; and (iii) the cut-off time that best discriminates individuals with hemiplegia from chronic stroke and healthy older adults.

**Methods:**

33 individuals with hemiplegia due to chronic stroke and 32 healthy elderly individuals participated in this cross-sectional study. The timed 180° turn test was administered along with other measures of stroke-specific impairment.

**Results:**

The timed 180° turn test demonstrated excellent intrarater, interrater, and test-retest reliability in individuals with hemiplegia from chronic stroke. The timed 180° turn test (times) significantly correlated with the Fugl-Meyer Assessment of the Lower Extremities (FMA-LE), affected ankle plantar flexion strength, the 5-Times-Sit-To-Stand test, the Berg Balance Scale (BBS), and the Timed Up and Go (TUG) test.

**Conclusion:**

The timed 180° turn test is a reliable clinical tool to assess the turning ability of subjects with hemiplegia from chronic stroke.

## 1. Introduction

Turning is a fundamental motor skill of mobility and is essential in the execution of many activities of daily living [1]. Basic functional activities like toileting require more than 2 turns for every 10 steps taken [2]. Complex tasks and smaller homes increase the amount of turning required [2, 3]. People with hemiplegia from chronic stroke often experience persistent impairments in strength, balance, and coordination of gait [4, 5] which impacts on their ability to safely change direction, negotiate obstacles, and turn [1].

Sedgman et al. [2] investigated and quantified the angles of turns that occur during 8 functional activities in a home environment. Examples of these activities include walking to the bedroom to put on shoes and socks and walking to the bathroom with simulated toileting. For the 8 functional activities investigated in their study, 86.6% of all turns occurred within the range of 30°–165° and 100% of turns were within the range of 30°–255° [2]. Although the 360° turn is one component of the Berg Balance Scale (BBS) [6] and has been shown to have excellent intrarater, interrater, and test-retest reliability in individuals with chronic stroke [7], the 180° turn is used frequently in functional activities and is therefore an important angle to investigate.

The mini-BESTest [8], the Figure-of-Eight Walk test [9], the Timed Up and Go (TUG) test [10], the Dynamic Gait Index [11], and the Six-Minute Walk Test [12] are outcome measures that incorporate a 180° turn but the turn occurs while walking. To enable turning while walking the body must reduce its forward momentum, rotate, and then accelerate into the new direction [13]. This presents a methodological problem in determining when the turn starts and finishes. These outcome measures are reliable and valid for use in subjects with stroke [14–18] but they do not independently assess the individuals' turning ability.

Walking and turning are common activities reported at the time of a fall in older adults [19] and falls during turning are 7.9 times more likely to result in a hip fracture than falls that occur during straight walking [20]. The ability to adapt gait and balance and perform a turn or change in direction safely is essential for independence within the home and community. A reliable, independent measurement tool to assess turning for people with hemiplegia from chronic stroke is required.

The objectives of the study were (i) to establish the intrarater, interrater, and test-retest reliability of the timed 180° turn test for times and number of steps for people with hemiplegia from chronic stroke, (ii) to investigate the concurrent validity of the timed 180° turn test by exploring correlations with other impairment and activity limitation measures, including the Fugl-Meyer Assessment of the Lower Extremities (FMA-LE), ankle dorsiflexion and plantar flexion strength, the 5-Times-Sit-To-Stand test, the BBS, the TUG test, and the Activities-specific Balance Confidence (ABC) Scale. This study was also designed to (iii) determine the cut-off times that best discriminate individuals with hemiplegia from chronic stroke from healthy older adults when completing the timed 180° turn test to either direction.

## 2. Methods

### 2.1. Participants

This was a cross-sectional study. Good intrarater reliability (intraclass correlation coefficient [ICC] = 0.828) has been reported for the number of steps taken to turn 180° in older adults [21]. Assuming that ICC values of stroke survivors are about 0.90, we determined that a sample size of 30 would be required to achieve 90% power to detect an ICC of 0.90 with a confidence level of 0.05.

Individuals with hemiplegia from chronic stroke were recruited from a local self-help group and were included if they (i) were ≥55 years of age; (ii) had hemiplegia due to a single stroke at least one year previously; (iii) were able to walk at least 10 m with or without a walking aide; (iv) had an Abbreviated Mental Test score ≥ 7 [22]; and (v) had a stable general medical condition. Individuals were excluded if they presented with neurological conditions other than hemiplegia or with other comorbid disabilities that would hinder proper assessment. Healthy older adults were recruited from local community centres using poster advertising and were included if they were ≥55 years old. Healthy subjects were excluded if they had uncontrolled diabetes or any neurologic or musculoskeletal condition that would affect mobility or interfere with the assessment procedure.

The study was approved by the ethics committee of The Hong Kong Polytechnic University and was conducted according to the guidelines of the Declaration of Helsinki. Written informed consent was obtained from all participants before commencement of the study.

### 2.2. Outcome Measurements

For the timed 180° turn test, a piece of coloured tape is placed on the floor to mark the starting position. Each subject was required to stand with arms by side and feet comfortably apart and pointing to the tape. All subjects were asked to turn 180° on the spot from a standing start position and within a designated area marked on the floor. Subjects were asked to turn “as fast as you can” and wore their usual footwear. Timing (with a stopwatch) was started from the word “GO” and stopped when the shoulders and feet were facing in the opposite direction. The number of steps taken to turn 180° were also counted. Each subject performed three turns to each direction with 1-minute rest break between trials.

The FMA-LE is a comprehensive quantitative measure of the motor impairment of the lower extremities following stroke [23]. Seventeen items that assess reflexes, movement, and coordination are scored on an ordinal scale from 0 to 2, with a total maximum score of 34 [24]. The FMA-LE is known to have high interrater (ICC = 0.89–0.95) and intrarater (ICC = 0.96) reliability when used with individuals with chronic stroke [25].

The maximum isometric contraction of the subjects ankle dorsiflexors and plantar flexors was measured bilaterally using the Nicholas hand-held dynamometer (model 01160) (supplier: Lafayette Instrument Co.) in a standardised testing position [26]. Hand-held dynamometry for the ankle dorsiflexors and plantar flexors has been shown to have good intra- and interrater reliability (ICCs ≥ .70) [26]. Each contraction was held for 3 seconds against the examiners' resistance and each muscle group was tested three times with a rest break of 1 minute between trials. The average of the 3 trials was used for data analysis.

Functional muscle strength of the lower extremities was assessed with the 5-Times-Sit-To-Stand test [27]. It has been shown to have excellent intrarater (ICC = 0.970–0.967), interrater (ICC = 0.999), and test-retest (ICC = 0.989–0.999) reliability in subjects with chronic stroke [28]. Time was recorded for the subjects to stand up and sit down as quickly as possible five times using a standardised testing protocol [28]. Subjects completed 3 trials with 1-minute rest between trials. The average of the 3 trials was used for data analysis.

Clinical balance was measured with the BBS [6]. The BBS measures balance performance during 14 functional tasks rated on a 0–4 ordinal scale, giving a maximum score of 56 [6]. The BBS has been shown to have excellent interrater (ICC = .98) and intrarater (ICC = .97) reliability in subjects with stroke [29].

Functional mobility was assessed by the TUG test [10]. The TUG test has been shown to have excellent test-retest reliability (ICC = 0.95) in subjects with chronic stroke [15]. Time was recorded for subjects to stand from a chair, walk forward 3 metres at their comfortable walking speed, turn around, walk back, and sit down on the chair [10]. All subjects completed 3 trials with 1-minute rest in between. The average of the 3 trials was used for data analysis.

The ABC Scale measures a subjects' confidence in performing sixteen tasks without falling [30]. The subject rates their confidence on a 0%–100% scale, with 0% representing no confidence and 100% representing complete confidence in performing the activity without losing balance [30]. An overall score is calculated by averaging the scores for all items and results in a single score out of 100%. The ABC Scale has good test-retest reliability (ICC = 0.85) in subjects with chronic stroke [31]. The Cantonese version of the ABC Scale was used and has been shown to be a reliable and valid tool for measuring self-perceived balance confidence in older Chinese adults [32].

### 2.3. Testing Procedures

**Figure 1:**
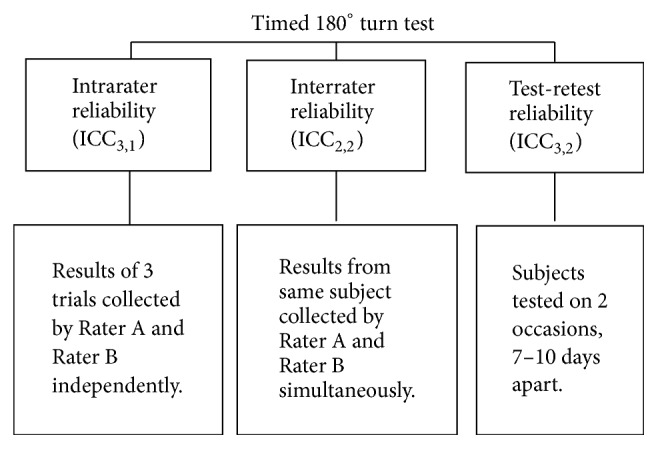
Schematic diagram of the data collection process for the timed 180° turn test. ICC_2,2_: intraclass correlation coefficient model 2,2; ICC_3,1_: intraclass correlation coefficient model 3,1; ICC_3,2_: intraclass correlation coefficient model 3,2.

The subjects with hemiplegia from chronic stroke were assessed on two separate occasions, 7 to 10 days apart. The interval of 7–10 days was chosen to avoid fatigue, learning, or memory effects for the subjects but also to avoid genuine change occurring during the study period [33]. The times and number of steps to complete the timed 180° turn test were recorded by 2 trained assessors simultaneously and independently. The data collection procedure is shown in Figure 1.

In addition to the timed 180° turn test, the subjects with hemiplegia from chronic stroke completed the FMA-LE, ankle dorsiflexor and plantar flexor muscle strength testing, the 5-Times-Sit-To-Stand test, the BBS, the TUG test, and the ABC Scale questionnaire. The outcome measures were completed in random order by drawing lots. Subjects were given a 2-minute rest break between outcome measures to avoid fatigue.

The healthy older adults completed the timed 180° turn test in one session. Their data was used to determine the timed 180° turn test cut-off times to distinguish healthy older adults from people with hemiplegia from chronic stroke.

### 2.4. Statistical Analysis

Data analysis was completed with SPSS software (version 23) (supplier: SPSS version 23.0; SPSS). The normality of the data and homogeneity of variances were tested using the Shapiro-Wilk test and Levene's test, respectively. Between-group differences in demographic data were assessed by the independent *t*-test and the Chi-square test. The within-group differences were assessed using the paired *t*-test for parametric data and the Wilcoxon signed-ranks test for nonparametric data.

The ICC was computed to measure the intrarater reliability (ICC_3,1_), interrater reliability (ICC_2,2_), and test-retest reliability (ICC_3,2_). Model 3 was chosen to establish that specific investigators were reliable in their data collection (rater is considered a fixed effect) and model 2 was chosen to generalise results to other raters with similar characteristics (rater and subjects are considered random effects).

The standard error of measurement (SEM) reflects the reliability and stability of the measurement instrument [33] and was calculated for the times and number of steps to turn 180° for each direction with the following formula: SEM = sd√1 − *r*, where sd is the standard deviation of the timed 180° turn test and *r* is the test-retest reliability coefficient.

The minimal detectable change with 95% confidence interval (MDC_95_) is calculated to determine how much change must occur in a variable to reflect true change [33]. The MDC_95_ for the times and number of steps to turn 180° for each direction was calculated with the following formula: MDC_95_ = 1.96 × SEM × √2.

Correlations between the timed 180° turn test times and number of steps (to both directions) and other outcome measures were quantified using Spearman rho due to nonparametric data. Six primary outcomes were chosen (FMA-LE, 5-Times-Sit-To-Stand test, BBS, TUG test, and affected ankle dorsiflexion and plantar flexion strength) and the *P* value for significant correlation was adjusted to 0.008 (0.05/6) after Bonferroni adjustment. The strength of correlation was classified into fair (*r *= 0.25–0.49), moderate to good (*r *= 0.50–0.75), and good to excellent (*r *≥ 0.75) [33].

Receiver operating characteristic curves were generated and by using Youden's index the timed 180° turn test cut-off times in seconds to both the affected and unaffected direction were obtained [33]. This cut-off time was used to distinguish the healthy older subjects from the subjects with hemiplegia from chronic stroke.

## 3. Results

Thirty-three subjects with hemiplegia due to chronic stroke (22 men [66.7%], 11 women [33.3%]) with a mean age of 60.18 ± 6.42 years and a mean time since stroke of 112.21 ± 51.30 months participated in this study. Thirty-two healthy control subjects (10 men [31.2%], 22 women [68.8%]) with a mean age of 61.84 ± 4.59 years were recruited. The subjects' demographics are summarized in Table 1. The hemiplegia group and healthy subject group were significantly different with regard to gender, height, weight, and body mass index.  Table 2 presents the mean values of all the other outcomes.

The mean times and number of steps taken to turn 180° for both subject groups are shown in Table 3. There was no significant difference in the times or number of steps for the hemiplegia group when turning towards the affected or unaffected side and for the healthy subjects when turning towards the right or left (Table 3). The hemiplegia group had significantly slower turn times and took significantly more steps to complete the timed 180° turn test than the healthy control group, regardless of turn direction (Table 4).

### 3.1. Reliability

The timed 180° turn test demonstrated excellent intrarater reliability for Rater 1 and Rater 2 for both turn directions, with ICCs ranging from 0.930 to 0.983 for times and 0.945 to 0.969 for number of steps (Table 5). Excellent interrater and test-retest reliability were found for both turn directions with ICCs ranging from 0.961 to 0.990 (Table 5). The MDC_95_ values for subjects turning towards the affected side were 0.62 seconds and 0.83 steps and towards the unaffected side were 0.64 seconds and 0.81 steps.

### 3.2. Correlation of the Timed 180° Turn Test with Other Outcome Measures

The details of the correlations are summarized in Table 6. The timed 180° turn test times (to affected and unaffected side) demonstrated significant correlations with the FMA-LE, affected ankle plantar flexion strength, the 5-Times-Sit-To-Stand test, the BBS, and the TUG test. The timed 180° turn test number of steps (to affected and unaffected side) demonstrated significant correlations with the BBS and the TUG. Following Bonferroni correction, the strength of the ankle dorsiflexors, the unaffected ankle plantar flexors, and the ABC Scale did not have a significant correlation to either the time taken or the number of steps taken to turn 180°.

### 3.3. Sensitivity and Specificity

**Figure 2:**
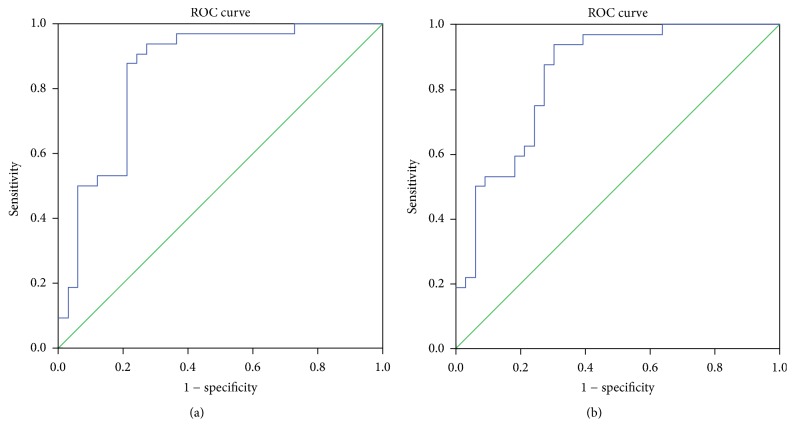
Timed 180° turn test ROC curves for times towards; (a) affected side (sensitivity 87.5%, specificity 78.8%, and AUC .851, *P* < 0.000) and (b) unaffected side (sensitivity 87.5%, specificity 72.7%, and AUC .846, *P* < 0.000).

A cut-off time of 2.59 seconds was found to best discriminate healthy subjects from those with hemiplegia towards both the affected side (area under the curve 0.851, sensitivity 87.5%, and specificity 78.8%) and unaffected side (area under the curve 0.846, sensitivity 87.5%, and specificity 72.7%). The receiver operating characteristic curves are presented in Figure 2.

## 4. Discussion

This is the first study to investigate the reliability and concurrent validity of the timed 180° turn test for individuals with hemiplegia from chronic stroke. It is also the first to determine the cut-off times of the timed 180° turn test best discriminating individuals with hemiplegia from chronic stroke and healthy older adults and to calculate the SEM and the MDC_95_.

### 4.1. Performance of the Timed 180° Turn Test

Turn direction (towards affected or unaffected side) did not have a significant effect on the timed 180° turn test times and number of steps for the subjects with hemiplegia from chronic stroke. This finding supports previous published studies. For subjects with stroke, turn direction does not have a significant effect on the time to complete the TUG test [34], the distance walked in the Six-Minute Walk Test [35], and the time and number of steps to complete a 360° turn [36]. It is hypothesised that patients with chronic stroke use compensatory strategies to adjust for the impairments and asymmetry between the affected and unaffected side and this adjustment results in similar turn performance to either direction [36].

Two types of turn strategy have been reported: the spin turn and the step turn [13]. The step turn is considered to be more stable due to the wide base of support [13] and because it requires less ankle coordination than the spin turn [37]. Whether individuals with hemiplegia from chronic stroke adopt the step turn strategy in turning 180° warrants further investigation through biomechanical studies.

Compared to the healthy control group, the hemiplegia group on average took 1.14 seconds longer to turn towards the affected side and 1.28 seconds longer to turn towards the unaffected side. This difference is greater than the calculated MDC_95_ (0.62 and 0.64 seconds, respectively). Similarly, the average number of steps taken to turn 180° for the hemiplegia group was 0.98 (towards the affected side) and 1.09 (towards the unaffected side) more than the healthy control group which is also greater than the calculated MDC_95_ (0.83 and 0.81 steps, respectively). Therefore, the difference in time and number of steps taken between the hemiplegia and healthy group to turn 180° is unlikely to be due to measurement error but is the result of a true difference in performance ability. This finding supports previous published studies reporting that subjects with stroke take significantly more time and more steps to turn 180° than healthy controls, independent of turn direction [38]. Individuals with hemiplegia from chronic stroke often experience residual impairments in strength, balance, and mobility [5] which could contribute to the differences in turning ability demonstrated.

### 4.2. Reliability

The TUG test has a 180° turn component and has been shown to have excellent test-retest reliability (ICC = 0.95) for individuals with chronic stroke [15]. For this study, detailed testing procedures, clear instructions to the subjects, and the raters adherence to standardised protocols may have contributed to the high intrarater, interrater, and test-retest reliability demonstrated.

### 4.3. Correlation of the Timed 180° Turn Test with Outcome Measures

Significant negative correlation was found between the FMA-LE and the time taken to turn 180° (to both the affected and unaffected side). The FMA-LE measures motor impairment after stroke by assessing reflexes, coordination, and isolated movement [24] and turning requires coordinated segmental movement of the pelvis and lower extremities [39]. Therefore, a strong correlation between these two variables would be expected.

Affected ankle plantar flexion strength demonstrated a significant negative correlation with the timed 180° turn test times to both directions. This is similar to the findings of Ng and Hui-Chan who reported that affected ankle plantar flexion strength had a significant negative correlation with the TUG test in subjects with chronic stroke (*ρ* = −0.860, *P* < 0.01) [15]. The ankle plantar flexors play an important role in propelling the body during a turn and it has been hypothesised that they play a greater role in moving the body during step turns compared to spin turns [37]. Following Bonferroni correction, the affected ankle dorsiflexors did not have a significant correlation to the times or number of steps to turn 180° for either turn direction. This is similar to the findings of Ng and Hui-Chan who reported that ankle dorsiflexion strength did not correlate with the TUG test in subjects with chronic stroke [15]. The lack of significant correlation between ankle dorsiflexion strength and the timed 180° turn test may be due to the dominant role of the ankle plantar flexors in forward propulsion [37].

The BBS scores demonstrated significant negative correlation with the timed 180° turn test times and number of steps. This may be the result of similarity between turning and 3 items within the BBS. Item 11 of the BBS (turn 360°), item 12 (place alternate foot on stool while standing unsupported), and item 14 (standing on one leg) have components of weight shifting, alternate stepping, and single support stance, which are also components of turning. The TUG test has a 180° turn component; therefore it is not surprising that this variable had a significant positive correlation to the timed 180° turn test times and number of steps taken to both directions. Subjects with better turning ability would be expected to perform well in both the TUG and 180° turn tests.

It is interesting to note that following Bonferroni correction the ABC Scale did not correlate significantly with the timed 180° turn test. The ABC Scale requires subjects to rate their balance confidence in performing 16 tasks without falling; therefore the subjects' perceived ability may not have a strong association with physical performance of turning measured by the timed 180° turn test [31]. In addition, the timed 180° turn test was conducted in our secure indoor laboratory which is different from the real-life environment of the ABC Scale.

Independent of turn direction, the time to turn 180° correlated with more outcome measures and consistently demonstrated a stronger correlation to those outcome measures than the number of steps taken to turn. One hypothesis for this finding is based on turn strategy. Without recording the type of strategy used, it is unknown whether significant heterogeneity may exist within the sample if some subjects were using a step turn and others were using a spin turn. This would reduce the sample size and power and hence its ability to detect a relationship between the number of steps taken to turn and measures of motor impairment and strength.

### 4.4. Sensitivity

The area under the curve for the timed 180° turn test times was 84.6% towards the unaffected side and 85.1% towards the affected side. A perfect test instrument would have an area under the curve (true positive rate) of 100% [33]. Based on these results, the timed 180° turn test is highly accurate (to either turn direction) in discriminating between the subject groups.

### 4.5. Study Limitations

The strategy and quality of the turn were not recorded as this would be too difficult for the raters to assess while also timing and counting the steps. By recording the turn strategy used by the subjects, the relationship between turn strategy, muscle strength, balance, and functional ability could be further investigated.

Caution should be exercised when interpreting the cut-off results due to the majority of hemiplegic subjects being male (66.7%) whereas the majority of the healthy subjects were female (68.8%). Previous research has shown that gender differences exist in lower extremity strength, functional mobility, and balance [40] which could have influenced the average performance of these 2 groups.

The results can only be generalised to subjects fulfilling the same selection criteria because of the small sample size. The sample size calculation was based on previous reliability findings [21], as the primary objective of this study is to investigate the reliability of timed 180° turn test in people with hemiplegia from chronic stroke. Future studies with larger samples are necessary to increase the generalizability of these results in people with hemiplegia from chronic stroke of different mobility levels.

## 5. Conclusion

In conclusion, the timed 180° turn test has excellent intrarater, interrater, and test-retest reliability in individuals with hemiplegia from chronic stroke. The timed 180° turn test (times) significantly correlated with the Fugl-Meyer Assessment of the Lower Extremities (FMA-LE), affected ankle plantar flexion strength, the 5-Times-Sit-To-Stand test, the Berg Balance Scale (BBS), and the Timed Up and Go (TUG) test.

The timed 180° turn test is a simple and reliable clinical tool to assess the turning ability of subjects with hemiplegia from chronic stroke.

## Figures and Tables

**Table 1:** Demographic characteristics of the 2 subject groups.

Characteristics	Hemiplegia	Healthy subjects	*P* (subjects with hemiplegia versus healthy subjects)
	Subjects (*N* = 33)	(*N* = 32)	
Age (y)	60.18 ± 6.42	61.84 ± 4.59	0.233
Gender (M/F)	22/11	10/22	0.004^*∗*^
Height (cm)	161.29 ± 7.03	157.25 ± 6.53	0.019^†^
Weight (kg)	66.81 ± 12.21	57.79 ± 7.41	0.001^*∗*^
Body mass index (kg/m^2^)	25.50 ± 3.20	23.35 ± 2.49	0.004^*∗*^
Months since stroke	112.21 ± 51.30	NA	NA

*Note*. Values are mean ± SD or as otherwise noted. M: male, F: female, NA: not applicable. ^*∗*^Significant difference at *P* < 0.01. ^†^Significant difference at *P* < 0.05.

**Table 2:** Mean values of outcome measures for subjects with hemiplegia (*N* = 33).

Assessment	Mean value ± SD
FMA-LE	23.8 ± 6.2
Affected ankle strength (kg)	
Dorsiflexion	9.5 ± 5.5
Plantar flexion	13.9 ± 5.7
Unaffected ankle strength (kg)	
Dorsiflexion	15.2 ± 3.3
Plantar flexion	18.8 ± 4.3
FTSTS (s)	19.2 ± 10.0
BBS	48.6 ± 4.4
TUG (s)	15.9 ± 6.3
ABC-C Scale	78.0 ± 15.6

*Note*. “C” indicates Chinese version of ABC.

**Table 3:** Mean times and number of steps for both subject groups.

Hemiplegia	Affected	Unaffected	*P *(turn towards affected versus unaffected side)
(*N* = 33)			
Time (s)	3.32 ± 1.23	3.46 ± 1.47	0.153
Steps	5.31 ± 1.54	5.42 ± 1.61	0.434

Healthy (*N* = 32)	Left	Right	*P* (turn towards left versus right)

Time (s)	2.18 ± .53	2.18 ± .51	0.988
Steps	4.39 ± .85	4.27 ± .96	0.139

*Note*. Values are mean ± SD. The mean values for the hemiplegia group were calculated from all the observations, including those from Rater 1 and Rater 2, Day 1 and Day 2.

**Table 4:** Mean times and number of steps for both subject groups.

	Hemiplegia subjects (*N* = 33)	Healthy subjects (*N* = 32)	*P* (subjects with hemiplegia versus healthy subjects)
Time (s)	Affected: 3.32 ± 1.23	2.18 ± .51	0.000^*∗*^
	Unaffected: 3.46 ± 1.47		0.000^*∗*^

Steps	Affected: 5.31 ± 1.54	4.33 ± .88	0.003^*∗*^
	Unaffected: 5.42 ± 1.61		0.003^*∗*^

*Note*. Values are mean ± SD. The mean values for the healthy group were calculated from all the right and left observations. ^*∗*^Significant difference at *P* < 0.01.

**Table 5:** Reliability of the timed 180° turn test for subjects with hemiplegia (*N* = 33).

Variable	Assessor	Turning direction	Day	Time	Number of steps
				ICC (95% CI)	ICC (95% CI)
Intrarater reliability (ICC_3,1_)	1	Affected	1	.930 (.875–.963)	.947 (.905–.972)
			2	.974 (.954–.986)	.945 (.902–.971)
		Unaffected	1	.983 (.970–.991)	.958 (.926–.978)
			2	.967 (.940–.982)	.969 (.945–.984)
	2	Affected	1	.971 (.949–.985)	.965 (.937–.981)
			2	.961 (.931–.980)	.950 (.911–.974)
		Unaffected	1	.973 (.952–.986)	.967 (.941–.983)
			2	.979 (.963–.989)	.963 (.934–.980)

Interrater reliability (ICC_2,2_)	1-2	Affected	1	.977 (.962–.987)	.979 (.965–.988)
			2	.977 (.963–.988)	.970 (.950–.983)
		Unaffected	1	.990 (.983–.994)	.983 (.973–.991)
			2	.985 (.975–.992)	.984 (.973–.991)

Test-retest reliability (ICC_3,2_)	1	Affected	1-2	.970 (.950–.983)	.961 (.939–.979)
		Unaffected	1-2	.985 (.975–.992)	.977 (.962–.987)
	2	Affected	1-2	.978 (.963–.988)	.975 (.958–.986)
		Unaffected	1-2	.982 (.970–.990)	.978 (.963–.988)

CI: confidence interval, ICC_2,2_: intraclass correlation coefficient model 2,2, ICC_3,1_: intraclass correlation coefficient model 3,1, and ICC_3,2_: intraclass correlation coefficient model 3,2.

**Table 6:** Correlations between the timed 180° turn test and other outcome measures.

Outcome measures	Affected side		Unaffected side	
	Time	Steps	Time	Steps
FMA-LE	**−.625** ^*∗*^	**−.460** ^*∗*^	**−.661** ^*∗*^	−.392
Affected ankle strength (kg)				
Dorsiflexion	−.346	−.153	−.418	−.121
Plantar flexion	**−.535** ^*∗*^	−.348	**−.547** ^*∗*^	−.292
Unaffected ankle strength (kg)				
Dorsiflexion	−.180	−.284	−.112	−.192
Plantar flexion	−.023	.136	−.001	.213
FTSTS	**.573** ^*∗*^	.395	**.560** ^*∗*^	.271
BBS	**−.649** ^*∗*^	**−.569** ^*∗*^	**−.722** ^*∗*^	**−.521** ^*∗*^
TUG	**.714** ^*∗*^	**.506** ^*∗*^	**.765** ^*∗*^	**.470** ^*∗*^
ABC-C Scale	−.363	−.281	−.344	−.205

*Note*. Values are Spearman rho (*ρ*). ^*∗*^Significant correlation after Bonferroni adjustment at a *P* value of 0.05/6 (*P* < 0.008). FMA-LE: Fugl-Meyer Assessment of the Lower Extremity, FTSTS: 5-Times-Sit-To-Stand, BBS: Berg Balance Scale, TUG: Timed Up and Go, and ABC-C Scale: Activities-specific Balance Confidence (Chinese version) Scale.
